# Supplementary material for: Identification of photoperiod-induced specific miRNAs in the adrenal glands of Sunite sheep (Ovis aries)
Source: Front Vet Sci. 2022 Jul 22;9:888207. doi: 10.3389/fvets.2022.888207 (PMC9354845; doi:10.3389/fvets.2022.888207)
Supplement: Supplementary file 1 [file Data_Sheet_1.ZIP › Supplementary file/miRDeep2 file.zip!/miRDeep2 file/Know_Structure/SPLP42A3/oar-mir-409.pdf]

miRBase precursor : oar-mir-409
Total read count : 14303
oar-miR-409-5p read count : 1963
oar-miR-409-3p read count : 12331
remaining reads : 9

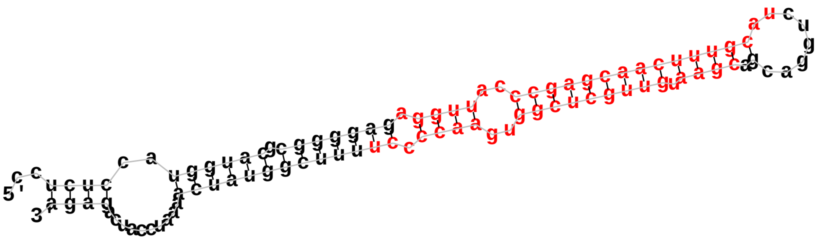

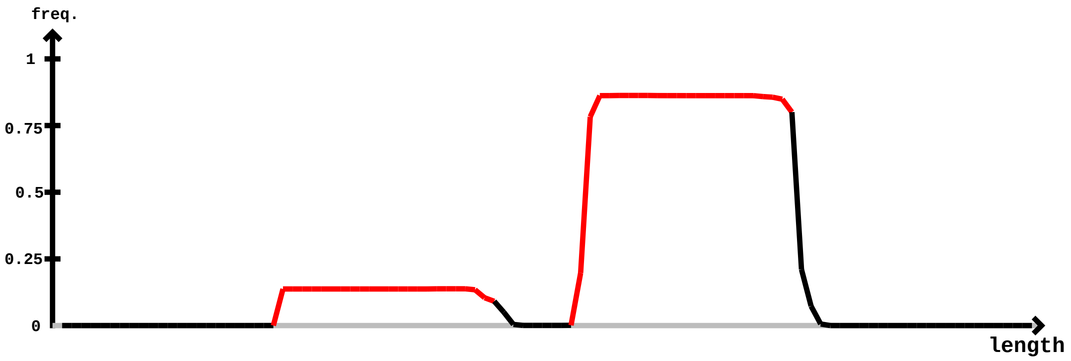

oar-miR-409-5p

oar-miR-409-3p

```
5'- ccucuccaugguacgcggggagagguuacccgagcaacuuugcaucuggacgacgaauguugcucggugaaccccuuuucgguaucaaaauccaucagaga   -3'   exp
    .((((...((((((.(((((((.((((..((((((((((((((........).)))).)))))))))..)))).)))))))))))))).........)))).    reads   mm   sample
    ....................agagguuacccgagcaacuuug...........................................................    1       0    seq
    ......................agguuacccgagcaacuuu............................................................    1       0    seq
    ......................agguuacccgagcaacuuug...........................................................    50      0    seq
    ......................agguuacccgagcaacuuugc..........................................................    428     0    seq
    ......................agguuacccgagcaacuuugca.........................................................    191     0    seq
    ......................agguuacccgagcaacuuugcau........................................................    586     0    seq
    ......................agguuacccgagcaacuuugcauc.......................................................    654     0    seq
    ......................agguuacccgagcaacuuugcaucu......................................................    52      0    seq
    ......................................uuugcaucuggacgacgaauguu........................................    7       0    seq
    ......................................uuugcaucuggacgacgaauguugcu.....................................    1       0    seq
    .......................................uugcaucuggacgacgaauguug.......................................    1       0    seq
    ....................................................acgaauguugcucgguga...............................    1       0    seq
    ....................................................acgaauguugcucggugaac.............................    4       0    seq
    ....................................................acgaauguugcucggugaacccc..........................    1       0    seq
    .....................................................cgaauguugcucggugaa..............................    1       0    seq
    .....................................................cgaauguugcucggugaac.............................    37      0    seq
    .....................................................cgaauguugcucggugaacc............................    12      0    seq
    .....................................................cgaauguugcucggugaaccc...........................    37      0    seq
    .....................................................cgaauguugcucggugaacccc..........................    242     0    seq
    .....................................................cgaauguugcucggugaaccccu.........................    1853    0    seq
    .....................................................cgaauguugcucggugaaccccuu........................    469     0    seq
    .....................................................cgaauguugcucggugaaccccuuu.......................    143     0    seq
    .....................................................cgaauguugcucggugaaccccuuuu......................    16      0    seq
    ......................................................gaauguugcucggugaac.............................    7       0    seq
    ......................................................gaauguugcucggugaacc............................    22      0    seq
    ......................................................gaauguugcucggugaaccc...........................    64      0    seq
    ......................................................gaauguugcucggugaacccc..........................    388     0    seq
    ......................................................gaauguugcucggugaaccccu.........................    5767    0    seq
    ......................................................gaauguugcucggugaaccccuu........................    1380    0    seq
    ......................................................gaauguugcucggugaaccccuuu.......................    689     0    seq
    ......................................................gaauguugcucggugaaccccuuuu......................    56      0    seq
    .......................................................aauguugcucggugaaccc...........................    5       0    seq
    .......................................................aauguugcucggugaacccc..........................    63      0    seq
    .......................................................aauguugcucggugaaccccu.........................    815     0    seq
```

```
                      oar-miR-409-5p
                                                       oar-miR-409-3p
ccucuccaugguacgcggggagagguuacccgagcaacuuugcaucuggacgacgaauguugcucggugaaccccuuuucgguaucaaaauccaucagaga

.......................................................aauguugcucggugaaccccuu........................    119    0    seq
.......................................................aauguugcucggugaaccccuuu.......................    121    0    seq
.......................................................aauguugcucggugaaccccuuuu......................    9      0    seq
.......................................................aauguugcucggugaaccccuuuuc.....................    1      0    seq
........................................................auguugcucggugaaccccu.........................    1      0    seq
.........................................................uguugcucggugaaccccuuu.......................    5      0    seq
.........................................................uguugcucggugaaccccuuuu......................    3      0    seq
```
